# Influence of Physical Activity, Physical Fitness, Age, Biological Maturity and Anthropometric Variables on the Probability of Suffering Lumbar, Neck and Shoulder Pain in Spanish Adolescents from the Region of Murcia

**DOI:** 10.3390/healthcare12181856

**Published:** 2024-09-15

**Authors:** Mario Albaladejo-Saura, Adrián Mateo-Orcajada, Lucía Abenza-Cano, Raquel Vaquero-Cristóbal

**Affiliations:** 1 Facultad de Deporte, Universidad Católica San Antonio de Murcia (UCAM), 30107 Murcia, Spain; mdalbaladejosaura@ucam.edu; 2 Research Group Movement Sciences and Sport (MS&SPORT), Department of Physical Activity and Sport Sciences, Faculty of Sport Sciences, University of Murcia, 30720 San Javier, Spain; raquel.vaquero@um.es

**Keywords:** back pain, healthy habits, adolescence, body composition, physical fitness

## Abstract

**Background:** Back pain in adolescents is a common injury, mainly affecting the lumbar, cervical and sometimes shoulder region. This has been related to various factors, such as lifestyle habits or physical capacity, but no previous research has shown conclusive results. The aims of this study was to analyze the risk of suffering lumbar, neck and shoulder pain according to anthropometric and physical fitness variables, physical activity level, age and biological maturity in adolescents, as well as the influence of sex in the study results. **Methods:** A descriptive cross-sectional study was performed, including a sample of 2015 adolescents (boys: *n* = 1006, mean age = 14.41 ± 1.35 years-old; girls: *n* = 1009, mean age = 14.48 ± 1.41 years-old). The participants underwent an anthropometric evaluation and physical fitness tests were carried out, including a 20 m shuttle run, a counter movement jump, a horizontal jump, a 20 m sprint and push-up tests, followed by the completion of lumbar, neck and shoulder pain questionnaires. **Results:** Higher values in age and peak height velocity (PHV) showed an increase in the risk of suffering lumbar, neck and shoulder pain (OR = 0.79–1.55; *p* = 0.000–0.025). The anthropometric variables related to adiposity showed an increase in the risk of suffering back pain, with significant incidence in the lumbar region (OR = 1.32–1.60; *p* = 0.000); while muscle mass showed a protective effect (OR = 0.59; *p* = 0.000). Regarding the fitness tests, a better physical fitness seemed to protect adolescents from suffering from the analyzed back pains in the general sample and in the boys sample (OR = 0.56–1.60; *p* = 0.000), while in the girls sample the influence of the physical fitness was less relevant. **Conclusions:** Both anthropometry and physical fitness may influence the occurrence of back pain in adolescents, with some variations in their importance according to sex.

## 1. Introduction

Back pain has a high prevalence in all populations, being the main cause of disability in the world [1]. Most studies have analysed lumbar pain in adult populations but there is evidence suggesting lumbar, neck and shoulder pain should also be analyzed in adolescence because the incidence and prevalence of pain in different areas of the spine are increasing in this population [2,3]. This type of pain can negatively affect the adolescent’s quality of life, health and participation in physical activities [4], having an important association between adolescence and adult back pain [5].

Previous research has shown that healthy habits in relation to physical activity and a sedentary lifestyle play a determining role in the prevalence of back pain in adolescents [6]. Current trends in the use of leisure time indicate that adolescents are increasingly spending more time engaging in sedentary activities rather than in physical exercise [7]. This fact is more evident since the COVID-19 pandemic, after which leisure or work time spent engaging in screen activities, including computer and internet use, watching television, mobile phone use, and playing videogames, have increased [8]. Physical inactivity weakens the muscles that support the spine, which can lead to a higher incidence of back pain, which together with the increased amount of time adolescents spend sitting, means that they may adopt postures that are prone to back pain [9]. Sedentary behaviors, on the other hand, are defined as activities with low energy expenditure, performed in rest positions [8]. In this sense, it has been shown that both physical inactivity and sedentary behavior increase the incidence or recurrence of back pain [8].

Lifestyles related to physical activity are also a determining factor in the changes in anthropometric variables. In the case of people who spend more time doing sedentary activities, a higher rate of obesity, being overweight and a greater accumulation of fat mass and less muscle mass are observed [10]. Some studies have linked certain anthropometric variables such as BMI, muscle mass or fat mass, waist and hips girths to back pain in adults [11,12]. In a study developed by Heuch et al. [12], an association between total adiposity and body mass with back pain was shown. On the other hand, Hussain et al. [11] have showed that fat mass, fat percentage, waist girth and BMI were related with back pain, with a dose-response association. In other study performed in adolescents, it was found that those students with lower back pain showed higher values of body mass, BMI and waist girth than those without pain [13]. However, not all the studies that explored the relation between anthropometric variables and back pain found the same results, as central adiposity was not related to back pain, neither was it to the waist/hip ratio [12]. Due to this controversy and the important relationship between the anthropometric factors and back pain, it is important to further investigate this topic.

Also, previous studies have shown that age and biological maturation play a role in the development of back pain in adolescents [14], referring a self-reported prevalence of back pain of 31%, 51.9% and 71.2% among children aged 10–13, 14–16 and 17–19 years, respectively [15]. Related to this, pubertal changes have been related to an increased risk of suffering musculoskeletal disorders, including back pain, attributing this to the rapid somatic growth that is observed during adolescence in both boys and girls [16,17]. However, although a relationship between maturation, growth and back pain is possible, the available evidence is not conclusive [16].

It is worth noting that most of the mentioned studies have included adult populations and have performed descriptive or longitudinal studies focused on addressing the differences between participants with and without back pain, specifically lumbar pain [11,12]. Other studies have included adolescent populations but have analysed the differences between groups with or without back pain, without analysing the relationship between the variables and the prevalence; or have included the variables related to quality of life, physical performance and anthropometry in an analytic way [4,6,9,13]. However, none of these studies have explored the risk of suffering back pain, including lumbar, neck and shoulder pain according to the age, biological maturation, anthropometric variables and physical fitness variables on a large adolescent population, analysing a general adolescent sample and dividing the analysis by sex.

Therefore, the objectives of the present study were: (1) to analyse the odd ratios of suffering lower back pain, neck pain and shoulder pain as a function of anthropometric variables and physical fitness variables, physical activity level, age and biological maturity in an adolescent population; and (2) to analyse whether there are differences between boys and girls in the risk of suffering lower back pain, neck pain and shoulder pain as a function of anthropometric variables and physical fitness variables, physical activity level, age and biological maturity.

Based on previous studies, it was hypothesised that those subjects who are older, whose maturational process is more advanced, with a higher amount of fat mass and lower muscle mass would be more likely to suffer from lumbar, neck and shoulder pain, while subjects who were more physically fit would be less likely to suffer from lumbar, neck and shoulder pain (H1). On the other hand, regarding the differences in risk when the sample was divided by sex, it was hypothesised that there would be differences in the risk of suffering lumbar, neck and shoulder pain as a function of age, maturation, physical activity level, anthropometric variables and physical fitness (H2).

## 2. Materials and Methods

### 2.1. Design

A cross-sectional study design was used, with a non-probabilistic convenience sample. The design was approved prior to initiation by the ethics committee of the Catholic University of Murcia (CE022102), following the declaration of Helsinki and in accordance with the code of the world medical association. In addition, the research design and protocol were established according to the recommendations of the STROBE declaration [18].

### 2.2. Participants

The calculations necessary to establish the sample size were performed with Rstudio 3.15.0 software (Rstudio Inc., Boston, MA, USA). The significance level was set at α = 0.05. The standard deviation (SD) was established based on body mass from previous studies (SD = 12.1) [13]. With an error (d) in the body mass of 0.75 kg, the required sample was 1000 subjects per group, aiming the study for a statistical power greater than 0.80, achieving a calculated power of 0.96, which is considered high.

A total of 2015 adolescents between the ages of 12 and 16 years old took part in the study (boys: *n* = 1006, mean age = 14.41 ± 1.35 years-old; girls: *n* = 1009, mean age = 14.48 ± 1.41 years-old). The schools with the largest number of adolescents enrolled in compulsory secondary education in the northern, southern, eastern and western areas of the Region of Murcia, Spain, were selected with the aim of obtaining a representative sample of adolescents from these areas. The school with the largest sample was contacted and, if they did not wish to participate, the next largest sample was contacted. Finally, the five schools with the largest sample were selected, following a methodology from previous research [19].

Once the school’s management team had agreed to participate, the teachers in charge of physical education were contacted. After obtaining their approval, a face-to-face meeting was held with the pupils and their parents in order to inform them of the procedure and the aims of the research. After this, any doubts were resolved and those adolescents interested in participating signed the informed consent form, as well as their parents.

Inclusion criteria were as follows: (a) aged between 12 and 16 years; (b) attending compulsory secondary education; (c) not presenting any incapacitating diseases that would prevent completion of the questionnaires or completion of the fitness tests.

### 2.3. Instruments

#### 2.3.1. Questionnaire Measurements

The Spanish version of the Physical Activity Questionnaire for Adolescents (PAQ-A) was used to measure the level of physical activity [20]. This questionnaire is a seven-day recall and consists of nine questions, the first eight of which are answered on a Likert scale from 1 to 5 (1: no physical activity; 5: a lot of physical activity). The arithmetic mean of the sum of these first eight items defines the level of physical activity. This score is between a minimum of 1 and a maximum of 5, with the cut-off point for determining that an adolescent has a moderate to vigorous level of physical activity being 2.75 [21]. The ninth question is answered dichotomously and allows us to find out whether in the last week they did not engage in normal physical activity for any reason. This questionnaire has an intraclass correlation coefficient for the final score of 0.71 [20].

The Spanish version of the Nordic Questionnaire was used to assess the musculoskeletal symptoms in the lumbar, neck and shoulders region [22,23,24]. This questionnaire probed more deeply into the analysis of the respective symptoms and contained questions on the duration of the symptoms over time and the previous seven days. The questionnaire analysed more thoroughly the severity of the symptoms in terms of their effect on daily activities and during leisure time, and in terms of the total duration of symptoms and sick leave during the preceding 12 months. This questionnaire included eight questions about lumbar pain, eight about neck pain, and nine about shoulder pain. All questions were dichotomous (yes or no) and closed multiple-choice questions with only one possible selection (Kuorinka et al., 1987 [23]). This questionnaire showed a very high validity and reliability (internal consistency: 0.81) and the intraclass correlation coefficient showed statistically significant values and an excellent correlation coefficient (r = 0.95) [24].

#### 2.3.2. Body Composition and Kinanthropometric Measurement

Body composition analysis was performed by three anthropometrists accredited by the International Society for the Advancement of Kinanthropometry (ISAK) (levels 2 to 4). Two basic measurements (body mass and height), three skinfolds (triceps, thigh and calf) and five girths (relaxed arm, waist, hips, thigh and calf) were taken according to the protocol standardised by the ISAK [25].

Each variable was measured twice, and a third measurement was made when the difference between the first two measurements exceeded 5% for skinfolds and 1% for all other measurements. The mean of the measured values when two measurements were taken, and the median when three measurements were taken, was used as the final value. Each subject’s measurements were performed by the same anthropometrist [25].

An inextensible tape, Lufkin W606PM (Lufkin, Missouri City, TX, USA), with an accuracy of 0.1 cm was used to measure girths; for skinfolds, a skinfold caliper (Harpenden, Burgess Hill, UK) was used with an accuracy of 0. 2 mm; for body mass, a TANITA BC 418-MA Segmental (TANITA, Tokyo, Japan) with an accuracy of 100 g was used; and for height, a SECA 213 stadiometer (SECA, Hamburg, Germany) with an accuracy of 0.1 cm was used. All instruments were previously calibrated.

Intra- and inter-rater technical errors of measurement (TEM) were calculated for a subsample. The intra-evaluator TEM was 0.04% for basic measurements; 1.35% for skinfolds and 0.03% for girths; and the inter-evaluator TEM was 0.05% for basic measurements; 1.99% for skinfolds and 0.07% for girths.

The final anthropometric values were used to calculate BMI, fat mass (%) [26], muscle mass [27], the sum of the three skinfolds (triceps, thigh and calf) and waist-to-hip ratio. For muscle mass, it was necessary to calculate corrected muscle perimeters. These were estimated by correcting limb girths for the corresponding skinfold, using a circular model of the limb cross-section and assuming that the thickness of the adipose tissue was half the thickness of the skinfold. Thus, corrected girths were calculated for the upper arm [relaxed arm girth-(π × triceps skinfold)], thigh [mid-thigh girth-(π × thigh skinfold)] and calf [calf girth-(π × calf skinfold)].

The maturity offset for adolescents was estimated using the sex-specific formula by Mirwald et al. [28]. Biological maturation was then calculated as: biological maturation = chronological age—maturity offset, having as the output variable the peak height velocity (PHV). This method has shown validity for estimating maturity offset against the gold standard, with R^2^ values of 0.92–0.89 for boys and 0.91–0.88 for girls.

#### 2.3.3. Physical Fitness Measurement

The 20 m shuttle run is an incremental test recognised for its high validity and reliability in assessing cardiorespiratory fitness in adolescents. The test concludes when the participant either reaches exhaustion or is unable to complete the 20 m distance before the beep sounds. The speed at which the participant stops the test is used to estimate maximal oxygen consumption (VO2max.) using the formula by Leger et al. [29].

The countermovement jump (CMJ) test was used to assess the explosive power of the lower limbs by measuring jump height. In this test, participants start from a standing position with their hands on their waists, bend their knees to a 90° angle, and then perform a full knee extension to achieve a maximum jump height, keeping their hands on their hips and their trunk fully extended during the flight phase. The test was conducted using a force platform with a sampling frequency of 200 Hz (MuscleLab, Stathelle, Norway) [30].

The 20 m sprint measured the minimum time taken by the participant to cover the specified distance. The participant started from a static position on the starting line and sprinted at maximum speed. Single-beamed photocells (Polifemo Light, Microgate, Italy) positioned at hip height were used, reducing the likelihood of being interrupted by arm movement to 4%, compared to a 60% probability when placed at chest height [31,32].

To measure upper limb strength, the push-up test was administered. Adolescents began in a prone position with the tips of their feet touching the floor and their arms placed at their sides with elbows bent at 90 degrees. From this position, participants lifted themselves off the floor until their arms were fully extended, maintaining a straight back and legs, and repeated this movement as many times as possible. The test concluded when the arms were no longer fully extended or when the participant reached exhaustion [33].

For the horizontal jump, the participant stood behind the starting line with their feet together, then propelled themselves powerfully and jumped forward as far as possible. Adolescents could use their arms to balance and gain momentum. The distance was measured from the starting line to the point where the back of the heel closest to the starting line landed on the mat or on the non-slip floor [33].

### 2.4. Procedure

All the tests were carried out in the selected high schools, using the class hour belonging to physical education and taking advantage of the classes and the covered sports pavilions to reduce the polluting variables of the environment as much as possible. All tests were performed on the same day.

First, the adolescents completed the PAQ-A questionnaire in a quiet environment, isolated from distracting noises. The researchers answered any questions but did not condition the adolescent’s response. Following this, anthropometric measurements were taken to assess body composition. Then, instructions on the proper execution of the CMJ, 20-m sprint, push-up, and horizontal jump tests were given to the adolescents for familiarization. Following this familiarization process, a progressive running warm-up with joint mobility exercises was performed for 10 min, and the CMJ, 20-m sprint, push-up and horizontal jump tests were conducted. The 20-m shuttle run test was performed after all other physical tests. Each physical fitness test was performed twice by each adolescent, with a 2-min rest between attempts and a 5-min rest between different tests, with the best value recorded, except for the 20-m shuttle run tests, which were performed only once.

The order of the physical fitness tests followed the recommendations of the National Strength and Conditioning Association (NSCA). To minimise interference in the results, 5-min rests were taken between tests to allow participants to recover from fatigue and meet the metabolic demands of each test [34]. This protocol for conducting physical fitness tests has been previously used in research with similar populations [35,36].

Four researchers with previous experience on the assessment of physical fitness tests oversaw the familiarization and assessment of these tests, with the same researchers being responsible for each test during all the measurements, in order to avoid inter-evaluator error in the assessments.

### 2.5. Statistical Analysis

The Kolmogorov–Smirnov and Mauchly’s W tests were used to evaluate the distribution and sphericity of the sample. The descriptive statistics mean, and standard deviation were calculated for the quantitative variables; and frequency and percentage were calculated for qualitative variables. The sample was analysed as a general group and divided by sex. To perform the Chi squared (χ^2^) and Odd Ratio tests, the continuous quantitative variables were categorised in relation to the group mean, dividing them into “greater than the mean” or “less than the mean”, in accordance with procedures previously followed in the literature [37,38]. The χ^2^ test was used to analyse differences between those participants suffering lumbar, neck and shoulder pain and those that declared no pain on categorised discrete quantitative variables and qualitative variables. Cramer’s V test (V) was used to calculate the magnitude of association between variables. It was then classified following the procedure of previous research considering V < 0.17 a small magnitude; V > 0.17 and V < 0.29 a medium magnitude; and V ≥ 0.29 a large magnitude, in line with previous research [39]. Finally, the odds ratio (OR) of suffering lumbar, neck or shoulder pain was calculated as a function of anthropometric and physical fitness variables. The results were reported as raw odds ratios (ORs) with 95% confidence intervals (CIs). The 95% CI of odds ratios was set to express the magnitude of the associations. Statistical analysis was performed using IBM SPSS Statistics (version 24.0). An error of *p* ≤ 0.05 was established.

## 3. Results

From the total sample, 886 (43.97%) suffered lumbar pain, 990 (49.17%) suffered neck pain and 542 (26.90%) suffered shoulder pain.

Table 1 and Table 2 show the frequency, mean and standard deviation (SD), χ^2^ and OR of those suffering lumbar pain in the general sample and the sample divided by sex, respectively. In both boys and girls, being younger was shown to decrease the chances of suffering lumbar pain (OR = 0.75–0.78; *p* = 0.028–0.047). Higher values in age and PHV showed to increase the risk of suffering lumbar pain in the general sample (OR = 0.79–1.55; *p* = 0.000–0.008) and in boys (OR = 1.41; *p* = 0.010). Regarding the anthropometric variables, higher values in body mass, BMI and the variables related to fat mass accumulation and distribution showed an increase in the risk of suffering lumbar pain in the general sample (OR = 1.32–1.60; *p* = 0.000) and both boys and girls separately (OR = 1.38–1.76; *p* = 0.000–0.044). Higher results in muscle mass percentage decreased the chances of suffering lumbar pain in the general sample (OR = 0.59; *p* = 0.000). In general, better results in the fitness tests decreased the chances of suffering lumbar pain in the general sample (OR = 0.56–1.60; *p* = 0.000). More specifically, higher values in VO2max in girls and better results in horizontal jump in boys decreased the risk of suffering lumbar pain (OR = 0.59–1.64; *p* = 0.000–0.006).

Table 3 and Table 4 show the frequency, mean and standard deviation (SD), χ^2^ and OR of suffering neck pain in the general sample and the sample divided by sex, respectively. Higher values in age and PHV showed an increase in the risk of suffering neck pain in the general sample (OR = 0.75–1.34; *p* = 0.001–0.002). Age also showed significant results in both boys and girls, with younger participants showing a lower risk of suffer neck pain (OR = 0.68–0.77; *p* = 0.003–0.043). Regarding the anthropometric variables, higher values in the variables related to fat mass accumulation and distribution showed an increase in the risk of suffering neck pain in the general sample (OR = 1.38–1.66; *p* = 0.000) and boys (OR = 1.52–1.73; *p* = 0.000–0.004). Higher values in height decreased the risk of suffering neck pain in the general sample (OR = 0.79; *p* = 0.007). Higher values in muscle mass percentage decreased the risk of suffering neck pain in the general sample (OR = 0.48; *p* = 0.000) and boys (OR = 0.56; *p* = 0.000). Regarding physical activity, being less physically active increased the risk of suffering neck pain (OR = 1.12; *p* = 0.008); while having better results in the physical fitness tests decreased the risk of suffering neck pain in the general sample (OR = 0.56–1.69; *p* = 0.000). Those with higher values of VO2max showed a lower risk of suffering neck pain both in boys and girls (OR = 0.68–0.77; *p* = 0.003–0.043). Regarding physical fitness, better results in the 20 m sprint and push-up tests reduced the chances of suffering neck pain in boys (OR = 0.67–1.64; *p* = 0.001–0.025), while no significant relations were observed in girls.

Table 5 and Table 6 show the frequency, mean and standard deviation (SD), χ^2^ and OR of suffering shoulder pain in the general sample and the sample divided by sex, respectively. A more advanced PHV was shown to increase the chances of suffering shoulder pain in the general sample (OR = 1.27; *p* = 0.025). Regarding the anthropometric variables, higher values in the variables related to fat mass accumulation and distribution were shown to increase the risk of suffering shoulder pain in the general sample (OR = 0.06–1.45; *p* = 0.000–0.033); while higher muscle mass percentage showed a decrease in the chances of suffering shoulder pain in the general sample (OR = 0.66; *p* = 0.000). In the case of the boys, higher values of body mass, height and the variables related to fat mass accumulation and distribution were related with a higher risk of suffering shoulder pain (OR = 1.48–1.87; *p* = 0.000–0.019). Regarding the physical fitness tests, better results showed a decrease in the risk of suffering shoulder pain in the general sample (OR = 0.58–0.65; *p* = 0.000). In the case of girls, only VO2max showed statistical significance, where higher values were associated with a lower risk of shoulder pain (OR = 0.67; *p* = 0.012). 

## 4. Discussion

The objectives of the present research were to analyse the risk of suffering lower back pain, neck pain and shoulder pain as a function of anthropometric and physical fitness variables, physical activity level, age and biological maturity in an adolescent population; and to analyse whether there are differences between boys and girls in this risk. It was found that being older and having a more advanced maturational process increased the risk of suffering lumbar and neck pain for the whole sample and for the boys and girls individually, as well as for also shoulder pain in the general sample. These results are consistent with those found in previous research [16]. Puberty is defined as an event that occurs during adolescence and is characterised by somatic changes, and significant musculoskeletal, physiological and sexual development, both in boys and girls [40]. Some authors have linked the fast changes that occur during the PHV with the risk of suffering back injuries and pain [41], finding an explanation in changes that adolescents experience at the anatomical and structural level in their bones and muscles at this stage in life [42]. However, a systematic review on the topic did not find relevant evidence to ensure a solid relationship between age, biological maturation and the risk of suffering back pain in adolescents [16]. Although more research is needed, the present research may provide information on the risk of lower back, neck and shoulder pain as a function of age and biological maturation.

The level of physical activity adolescents engage in also seems to influence the risk of lower back, neck and shoulder pain. In the present investigation it was found that in the overall sample, the less active subjects had a slightly higher risk of lumbar and neck pain, but not shoulder pain. When the groups of boys and girls were analysed, physical activity was found to have no influence on any of the types of pain analysed. A recent systematic review and meta-analysis carried out by Mahdavi et al. [8] indicated that the risk of suffering back pain was higher between the adolescents who had sedentary behaviours and who did not reach the daily physical activity recommendations, what may contrast with the results obtained in this research. However, another systematic review, carried out by Kedra et al. [14], proposed that the relationship between physical activity and back pain might be “U” shaped, meaning that the most active and most sedentary populations are more at risk than those with levels of activity close to the recommended amount. In this study, the PAQ-A questionnaire was used to assess the level of activity of the participants, with 1 being the minimum score and 5 being the maximum and all subjects with a score over 2.75 being considered active [21]. In this case, the groups comprising participants with and without pain in the different regions of the back were close to the average level of physical activity, slightly below those with back pain, which could explain the low relationship found between the level of physical activity and the risk of suffering back pain.

When analysing the risk of suffering lumbar, neck and shoulder pain in relation to anthropometric variables, the results showed that higher values in body mass, BMI, waist and hips girth, ∑3 skinfolds and fat mass percentage showed an increase in the risk of suffering lumbar pain; higher values in hips girth, ∑3 skinfolds and fat mass percentage showed an increase in the risk of suffering neck pain; and higher values in BMI, hips girth and ∑3 skinfolds showed an increase in the risk of suffering shoulder pain. These findings are in line with previous research, which has found an association between back pain and variables measuring both the amount of fat mass and measures of fat mass distribution, such as waist girth, hip girth and waist/hip ratio [43]. In this sense, excess body weight attributable to an increase in fat mass and the central distribution of fat mass could lead to a structural overload on the back, leading to a greater risk of suffering pain, and this has even been observed independently of the practice of physical exercise, which is considered a protective factor [44]. When the sample was divided by sex, the same tendency was observed regarding lumbar pain, but in the case of neck pain, the central adiposity variables only increased the risk of suffering neck pain in the boys. This may be due to the differences in the fat mass distribution that occur during adolescence due to the hormonal environment of boys and girls [45]. Women store more fat in the gluteal-femoral region, whereas men store more fat in the abdominal area [45]. As there is an association between central adiposity and the risk of back pain [43], the androgenic distribution of fat mass could explain why it is a more determinant factor to identify the risk of suffering back pain in boys than in girls.

An important finding from this research was that muscle mass percentage was shown to decrease the risk of suffering lumbar, neck and shoulder pain. This is because an adequate amount and strength of muscle mass helps with the maintenance of posture, including spinal alignment and balance [46], and contributes to the reduction of shear forces in the joints, improving stabilization [47]. Because of these characteristics, muscle mass may assume an important role in reducing back pain [48]. However, the protective factor of muscle mass was less relevant when the sample was divided by sex. Increased muscle mass is related to strength training [34]. However, the maturation process that happens during puberty leads to an increase in steroid hormones in both boys and girls that causes them to increase muscle mass, with a marked effect in the boys, so this variable will also increase whether or not they practice physical activity or have pain in the area [49], which could be a confounding factor. However, further research should explore the role of muscle mass in boys and girls in longitudinal designs to elucidate its relationship with back pain.

Regarding the physical fitness results, in the general sample, better results in the tests were found as an indicator of lower pain risk in the three areas analysed. When the sample was divided by sex, the same trend was observed, although the significance of the results was lower. In the case of lower back pain, only better results in VO2max in girls and in horizontal jumping in boys were indicative of lower risk; in the case of neck pain, better results in VO2max in girls and in all tests, except horizontal jumping, in boys were indicative of lower risk; and in the case of shoulder pain, only better results in VO2max in girls were indicative of lower risk. Health-related physical fitness comprises cardiorespiratory fitness and muscle strength/power [50]. One of the most popular measures of cardiorespiratory fitness is VO2max, measured indirectly through the 20 m shuttle run test [29]. Previous studies have reported an association between better levels of cardiorespiratory fitness and less risk of suffering back pain in children [51], which is in line with the findings of this study. Other important factors in physical fitness in adolescents is muscle strength/resistance and power [52]. In this research, better results in muscular strength/resistance tests (push-up test) and muscular power (sprint, CMJ and horizontal jump) were associated with a lower risk of suffering back pain. In the adult population, strengthening exercises seem to reduce back pain and increase the functionality of the spine [53], while lower limb power has been associated with lower lumbar pain [54]. However, despite all that, the relation between muscle strength/resistance and power with the prevalence of back pain remains unclear, as other studies have found no association between back muscle strength and lower back pain in adolescents [55], nor between performance when doing push-ups and LBP in the child and adolescent populations [56]. Therefore, future research should investigate this topic further by conducting longitudinal studies to further investigate back pain with strength training-based interventions.

Regarding the H1, it has been confirmed that, in the general sample, those subjects who were older, with a more advanced maturational process, less physically active and with greater results in the variables related to fat mass and central distribution of fat mass had a higher risk of suffering back pain in the regions analysed, while muscle mass and better results in the fitness tests decreased the risk of pain. Regarding the H2, it was confirmed that there are differences in the influence that the different variables have in the risk of suffering back pain according to sex. While age and fat mass related variables remained relevant for both boys and girls, maturational process and the results of physical fitness tests were more relevant in the boys sample, and the value of the VO2max was more determinant in the girls sample.

One of the strengths of this research is the number of participants included, which reached a total of 2015 adolescents from different schools with various environments. However, the extrapolation of the results found should be taken with care, as the geographical distribution of the sample, being all the participants from the south-east of Spain, might make the results extendable only to populations with similar characteristics. Other strengths of the study include the comprehensive analysis conducted into the physical and anthropometric characteristics of participants, and being able to identify which of these variables were more related to the risk of suffering back pain. However, despite these results, the present research is not without limitations. One of the main limitations is the cross-sectional design of the study, which prevents the establishment of causal relationships between the variables studied and the risk of suffering back pain in the different regions [57]. In order to establish the possible causality between the different variables explored in this study and the risk of suffering back pain, long-term randomised experimental studies should be carried out in which interventions are made on the different variables to observe the changes in back pain [57]. Previous research has shown the relationship between back pain in adolescence and back pain in adulthood [5]. Therefore, future research should aim to carry out studies of the odd ratio of suffering some type of back pain as a function of age, biological maturation, level of physical activity, anthropometric variables and physical fitness with a longitudinal design. On the other hand, back pain plays a fundamental role in patients’ perceived quality of life [58]. In future research, quality of life related to back pain should be addressed. Another limitation of this research was that physical activity level was self-reported, setting the cutoff point to consider subjects active or sedentary in a punctuation of 2.75, resulting in small differences between active and sedentary groups. On the other hand, self-reporting physical activity may introduce a risk of bias in paediatric populations, even though some research has observed a high level of agreement between the subjective and objective tools for registering physical activity [59]. To reduce this risk of bias, future research should use more than one instrument to register physical activity, including accelerometery or heart rate registry, as they are the most used field methods [59]. Another possible bias risk might be the categorization of the continuous variables according to the group mean, considering the possibility of losing information. Notwithstanding, this categorization was made based on previous studies that performed similar statistical tests, both in populations with similar characteristics to the present one [38] as well as in different populations [37]. Nevertheless, future research could analyse the risk of suffering back pain in adolescents by dividing the sample according to the quartiles or deciles of the continuous variables.

In the present study a large number of variables have been analysed, both related to physical condition and kinanthropometric variables. However, there are some variables which can also show an influence on back pain in adolescents and which have not been taken into account, such as the lumbopelvic dynamics, the strength-resistance of core stabilizing muscles and the sagittal disposition of the spine [13], or hamstrings extensibility [60]. For this reason, future lines of research should include the assessment of variables such as physical activity with an objective tool; diet, because its relation to body composition; the influence of back stabilization muscles and back dynamics; and hamstrings extensibility.

Since the present study found that having poorer physical fitness, lower muscle mass and higher fat mass were associated with a greater likelihood of back pain, future lines of research should specifically analyse how interventions based on improving students’ physical fitness, aimed to increase cardiovascular capacity and strength as main factors, and body recomposition, including an increase in muscle mass and the reduction of the fat mass, affect back pain in longitudinal experiments, while controlling the possible confounding factors and the effect that this interventions may have in the psychological needs and quality of life perceptions of adolescents.

The practical applications of this study to reduce the risk of adolescents suffering lower back, neck or shoulder pain could be directed towards the implementation of systematised physical exercise programs, from schools or via extracurricular activities with the appropriate duration in time, including strength training, cardiovascular training and even the implementation of healthy diet habits, with the objective of increasing the level and intensity of physical activity practiced in this age group, since it has been seen that having a higher level of physical health, both in cardiovascular and strength tests, was a protective factor against back pain. On the other hand, achieving a body composition that increases muscle mass and reduces fat mass could also have a beneficial effect on lowering the risk of suffering this type of pain.

## 5. Conclusions

Almost half of the sample included in this study suffered from lumbar, neck or shoulder pain. Age, biological maturation, physical activity level, anthropometry and physical fitness may influence the occurrence of lumbar, neck and shoulder pain in adolescents, with some variations in their importance according to sex. In general terms, older adolescents and those whose maturational process is more advanced have a higher risk of suffering back pain. Physical activity levels did not have a determinant role in the risk of suffering back pain. Regarding the anthropometry, higher values in those variables related to adiposity and central distribution of fat mass were related to an increased risk of suffering back pain, while higher values in muscle mass percentage decreased the risk. Regarding physical fitness, higher values in the tests performed indicated a lower risk of suffering back pain in the overall sample, with particular relevance for the VO2max value when the sample was divided by sex.

## Figures and Tables

**Table 1:** Odd ratio of suffering lumbar pain according to fitness and anthropometric variables in the general sample.

Variable	Group (*n* = 2015)		χ^2^	*p*	Cramer’s V	OR	95% CI
	Not Lumbar Pain (*n* = 1129)	Lumbar Pain (*n* = 886)					
	Mean ± SD	Mean ± SD					
Age (years)	14.27 ± 1.35	14.65 ± 1.39	7.00	0.008	0.06	0.79	(0.66–0.94)
Physical activity level	2.73 ± 0.66	2.64 ± 0.64	6.67	0.010	0.06	1.11	(1.02–1.20)
BMI (kg/cm^2^)	20.89 ± 3.75	21.67 ± 3.91	12.17	0.000	0.08	1.15	(1.06–1.25)
Body mass (kg)	55.82 ± 12.69	58.20 ± 12.70	15.76	0.000	0.09	1.43	(1.20–1.71)
Height (cm)	163.01 ± 9.29	163.62 ± 8.80	1.23	0.268	0.02	1.04	(0.97–1.13)
Waist girth (cm)	69.16 ± 8.82	69.99 ± 8.64	9.39	0.002	0.07	1.32	(1.11–1.58)
Hips girth (cm)	89.43 ± 8.83	92.34 ± 8.98	40.82	0.000	0.14	1.78	(1.49–2.13)
∑3 skinfolds (mm)	48.42 ± 24.49	55.61 ± 26.03	26.56	0.000	0.12	1.60	(1.34–1.91)
Fat mass (% Slaughter)	21.35 ± 9.85	24.14 ± 10.25	24.99	0.000	0.11	1.57	(1.32–1.88)
Muscle mass (% Poortmans)	34.03 ± 5.48	32.18 ± 5.64	33.49	0.000	0.13	0.59	(0.49–0.71)
Waist/hip ratio	0.77 ± 0.06	0.76 ± 0.05	22.92	0.000	0.11	0.65	(0.54–0.77)
PHV (years)	0.24 ± 2.30	0.58 ± 2.59	21.07	0.000	0.10	1.55	(1.28–1.86)
20 m shuttle run test	4.19 ± 2.12	3.73 ± 2.05	22.61	0.000	0.10	0.65	(0.54–0.77)
VO2max (Legger’s formula)	38.94 ± 6.50	36.52 ± 6.97	40.22	0.000	0.14	0.56	(0.47–0.67)
CMJ (cm)	24.69 ± 6.54	23.58 ± 6.73	23.42	0.000	0.11	0.64	(0.54–0.77)
Sprint 20 m (s)	3.90 ± 0.42	4.00 ± 0.42	27.35	0.000	0.12	1.60	(1.34–1.91)
Push-up test (reps/min)	7.81 ± 9.78	6.08 ± 9.02	19.94	0.000	0.10	0.63	(0.51–0.77)
Horizontal jump (m)	1.46 ± 0.37	1.41 ± 0.39	4.44	0.035	0.05	1.34	(1.02–1.76)

BMI: Body mass index; ∑3 skinfolds: Sum of three skinfolds; PHV: Peak height velocity; CMJ: Counter movement jump.

**Table 2:** Odd ratio of suffering lumbar pain according to fitness and anthropometric variables in the sample divided by sex.

Variable	Boys (*n* = 1006)							Girls (*n* = 1009)						
	Not Lumbar Pain (*n* = 659)	Lumbar Pain (*n* = 347)	χ^2^	*p*	Cramer’s V	OR	95% CI	Not Lumbar Pain (*n* = 470)	Lumbar Pain (*n* = 539)	χ^2^	*p*	Cramer’s V	OR	95% CI
	Mean ± SD	Mean ± SD						Mean ± SD	Mean ± SD					
Age (years)	14.30 ± 1.34	14.63 ± 1.36	4.81	0.028	0.07	0.75	(0.57–0.97)	14.24 ± 1.38	14.67 ± 1.41	3.96	0.047	0.07	0.78	(0.61–1.00)
Physical activity level	2.86 ± 0.65	2.84 ± 0.60	0.54	0.464	0.02	0.91	(0.69–1.18)	2.54 ± 0.62	2.51 ± 0.63	2.16	0.142	0.04	1.21	(0.94–1.57)
BMI (kg/cm^2^)	21.02 ± 3.98	21.60 ± 3.90	5.78	0.016	0.07	1.38	(1.06–1.80)	20.70 ± 3.40	21.71 ± 3.92	6.93	0.008	0.08	1.41	(1.09–1.82)
Body mass (kg)	58.22 ± 13.99	61.47 ± 14.06	14.54	0.000	0.12	1.67	(1.28–2.17)	52.46 ± 9.65	56.07 ± 11.25	16.57	0.000	0.13	1.73	(1.33–2.26)
Height (cm)	165.95 ± 9.99	168.01 ± 10.00	8.86	0.003	0.09	1.53	(1.15–2.02)	158.89 ± 6.22	160.77 ± 6.51	9.77	0.002	0.10	1.54	(1.17–2.02)
Waist girth (cm)	71.44 ± 9.28	72.68 ± 8.50	17.63	0.000	0.13	1.76	(1.35–2.29)	65.96 ± 6.99	68.23 ± 8.28	8.93	0.003	0.10	1.51	(1.15–1.98)
Hips girth (cm)	89.04 ± 9.56	91.42 ± 9.38	13.96	0.000	0.12	1.65	(1.27–2.14)	89.97 ± 7.66	92.94 ± 8.67	19.70	0.000	0.14	1.76	(1.37–2.26)
∑3 skinfolds (mm)	41.60 ± 24.28	43.34 ± 24.73	0.90	0.343	0.03	1.15	(0.86–1.54)	58.02 ± 21.39	63.59 ± 23.67	6.52	0.011	0.08	1.39	(1.08–1.78)
Fat mass (% Slaughter)	18.69 ± 10.10	19.69 ± 10.76	0.21	0.645	0.01	1.07	(0.81–1.42)	25.07 ± 8.16	27.04 ± 8.77	9.49	0.002	0.10	1.49	(1.15–1.91)
Muscle mass (% Poortmans)	37.38 ± 4.28	37.30 ± 4.44	0.26	0.608	0.01	0.92	(0.65–1.28)	29.31 ± 2.93	28.84 ± 3.38	4.05	0.044	0.06	1.59	(1.01–2.50)
PHV (years)	−0.15 ± 2.22	−0.12 ± 2.55	6.58	0.010	0.08	1.41	(1.08–1.83)	0.77 ± 2.31	1.03 ± 2.51	0.00	0.997	0.01	1.00	(0.73–1.36)
20 m shuttle run test	4.99 ± 2.16	5.09 ± 2.12	0.00	0.993	0.01	1.00	(0.76–1.31)	3.02 ± 1.41	2.80 ± 1.38	3.31	0.069	0.06	0.75	(0.56–1.02)
VO2max (Legger’s formula)	41.09 ± 6.43	40.24 ± 7.19	0.76	0.383	0.03	0.88	(0.67–1.17)	35.89 ± 5.26	34.08 ± 5.63	13.45	0.000	0.11	0.59	(0.45–0.79)
CMJ (cm)	27.09 ± 6.64	27.90 ± 7.13	1.35	0.245	0.04	0.86	(0.66–1.11)	21.25 ± 4.57	20.82 ± 4.72	3.20	0.074	0.06	0.77	(0.57–1.03)
Sprint 20 m (s)	3.74 ± 0.37	3.73 ± 0.38	0.40	0.527	0.02	1.10	(0.82–1.48)	4.13 ± 0.37	4.17 ± 0.36	2.52	0.113	0.01	1.24	(0.95–1.62)
Push-up test (reps/min)	11.42 ± 10.77	11.90 ± 10.57	2.06	0.151	0.04	0.82	(0.63–1.07)	2.67 ± 4.65	2.55 ± 5.51	0.00	0.986	0.01	1.00	(0.65–1.54)
Horizontal jump (m)	1.60 ± 0.36	1.57 ± 0.43	7.67	0.006	0.09	1.64	(1.15–2.33)	1.29 ± 0.31	1.31 ± 0.34	2.44	0.118	0.05	1.45	(0.91–2.31)

BMI: Body mass index; ∑3 skinfolds: Sum of three skinfolds; PHV: Peak height velocity; CMJ: Counter movement jump.

**Table 3:** Odd ratio of suffering neck pain according to fitness and anthropometric variables in the general sample.

Variable	Group (*n* = 2015)		χ^2^	*p*	Cramer’s V	OR	95% CI
	Not Neck Pain (*n* = 1025)	Neck Pain (*n* = 990)					
	Mean ± SD	Mean ± SD					
Age (years)	14.34 ± 1.31	14.54 ± 1.44	10.31	0.001	0.07	0.75	(0.63–0.89)
Physical activity level	2.74 ± 0.65	2.64 ± 0.65	7.05	0.008	0.06	1.12	(1.03–1.22)
BMI (kg/cm^2^)	21.00 ± 3.66	21.47 ± 4.01	1.52	0.218	0.03	1.06	(0.97–1.16)
Body mass (kg)	56.55 ± 12.31	57.16 ± 13.18	0.13	0.719	0.01	0.98	(0.90–1.07)
Height (cm)	163.61 ± 9.49	162.91 ± 8.65	7.25	0.007	0.06	0.79	(0.66–0.94)
Waist girth (cm)	69.32 ± 8.29	69.72 ± 9.21	0.03	0.871	0.01	1.01	(0.92–1.10)
Hips girth (cm)	89.83 ± 8.72	91.58 ± 9.23	13.18	0.000	0.08	1.38	(1.16–1.65)
∑3 skinfolds (mm)	48.06 ± 24.94	55.37 ± 25.79	29.30	0.000	0.12	1.63	(1.37–1.95)
Fat mass (% Slaughter)	21.21 ± 10.10	24.02 ± 10.01	31.79	0.000	0.12	1.66	(1.39–1.98)
Muscle mass (% Poortmans)	34.27 ± 5.53	32.09 ± 5.54	64.24	0.000	0.18	0.48	(0.40–0.58)
PHV (years)	0.29 ± 2.33	0.48 ± 2.54	10.04	0.002	0.07	1.34	(1.12–1.61)
20 m shuttle run test	4.31 ± 2.19	3.65 ± 1.95	39.41	0.000	0.14	0.56	(0.47–0.68)
VO2max (Legger’s formula)	39.09 ± 6.71	36.65 ± 6.68	42.24	0.000	0.14	0.56	(0.47–0.67)
CMJ (cm)	25.01 ± 6.80	23.38 ± 6.39	29.50	0.000	0.12	0.61	(0.51–0.73)
Sprint 20 m (s)	3.89 ± 0.42	4.00 ± 0.42	34.51	0.000	0.13	1.69	(1.42–2.02)
Push-up test (reps/min)	8.51 ± 10.40	5.55 ± 8.18	40.47	0.000	0.14	0.52	(0.42–0.64)
Horizontal jump (m)	1.49 ± 0.41	1.39 ± 0.35	1.55	0.214	0.03	1.19	(0.90–1.56)

BMI: Body mass index; ∑3 skinfolds: Sum of three skinfolds; PHV: Peak height velocity; CMJ: Counter movement jump.

**Table 4:** Odd ratio of suffering neck pain according to fitness and anthropometric variables in the sample divided by sex.

Variable	Boys (*n* = 1006)							Girls (*n* = 1009)						
	Not Neck Pain (*n* = 607)	Neck Pain (*n* = 399)	χ^2^	*p*	Cramer’s V	OR	95% CI	Not Neck Pain (*n* = 416)	Neck Pain (*n* = 593)	χ^2^	*p*	Cramer’s V	OR	95% CI
	Mean ± SD	Mean ± SD						Mean ± SD	Mean ± SD					
Age (years)	14.34 ± 1.30	14.52 ± 1.42	9.04	0.003	0.09	0.68	(0.52–0.87)	14.34 ± 1.33	14.56 ± 1.46	4.08	0.043	0.06	0.77	(0.60–0.99)
Physical activity level	2.85 ± 0.64	2.86 ± 0.62	0.13	0.720	0.01	0.95	(0.74–1.23)	2.57 ± 0.62	2.49 ± 0.63	1.47	0.225	0.04	1.18	(0.90–1.53)
BMI (kg/cm^2^)	20.80 ± 3.66	21.86 ± 4.31	14.08	0.000	0.12	1.64	(1.26–2.12)	21.29 ± 3.64	21.20 ± 3.78	3.81	0.051	0.06	0.78	(0.60–1.00)
Body mass (kg)	58.07 ± 13.21	61.23 ± 15.19	7.87	0.005	0.09	1.44	(1.12–1.86)	54.31 ± 10.48	54.42 ± 10.83	1.79	0.181	0.04	0.84	(0.64–1.09)
Height (cm)	166.37 ± 10.26	167.05 ± 9.73	0.64	0.423	0.02	1.11	(0.86–1.45)	159.56 ± 6.35	160.13 ± 6.50	0.27	0.601	0.02	0.93	(0.71–1.22)
Waist girth (cm)	70.82 ± 8.60	73.43 ± 9.49	17.69	0.000	0.13	1.73	(1.34–2.24)	67.09 ± 7.29	67.22 ± 8.12	2.23	0.135	0.05	0.81	(0.62–1.07)
Hips girth (cm)	88.92 ± 8.93	91.26 ± 10.34	11.87	0.001	0.11	1.57	(1.21–2.03)	91.17 ± 8.23	91.80 ± 8.41	0.60	0.440	0.02	1.10	(0.86–1.42)
∑3 skinfolds (mm)	39.68 ± 22.71	46.47 ± 27.45	8.27	0.004	0.09	1.52	(1.14–2.01)	60.42 ± 22.88	61.38 ± 22.74	1.55	0.213	0.04	1.17	(0.91–1.51)
Fat mass (% Slaughter)	17.98 ± 9.75	20.76 ± 11.16	11.02	0.001	0.10	1.59	(1.21–2.08)	25.98 ± 8.63	26.21 ± 8.49	1.98	0.160	0.04	1.20	(0.93–1.55)
Muscle mass (% Poortmans)	37.63 ± 4.09	36.84 ± 4.78	12.14	0.000	0.10	0.56	(0.41–0.78)	29.30 ± 3.09	28.89 ± 3.24	0.27	0.605	0.02	1.13	(0.72–1.76)
PHV (years)	−0.18 ± 2.32	−0.08 ± 2.37	2.33	0.127	0.05	1.22	(0.95–1.57)	0.98 ± 2.17	0.86 ± 2.58	1.67	0.197	0.04	0.81	(0.59–1.11)
20 m shuttle run test	5.19 ± 2.17	4.76 ± 2.07	6.68	0.010	0.07	0.71	(0.55–0.92)	2.98 ± 1.41	2.86 ± 1.39	2.01	0.157	0.04	0.80	(0.59–1.09)
VO2max (Legger’s formula)	41.49 ± 6.54	39.79 ± 6.75	5.03	0.025	0.07	0.74	(0.56–0.96)	35.54 ± 5.22	34.50 ± 5.71	6.11	0.013	0.08	0.70	(0.53–0.93)
CMJ (cm)	27.60 ± 6.69	27.01 ± 7.02	1.16	0.283	0.03	1.19	(0.86–1.65)	21.16 ± 4.86	20.93 ± 4.50	3.52	0.061	0.06	0.75	(0.56–1.01)
Sprint 20 m (s)	3.71 ± 0.36	3.78 ± 0.39	11.35	0.001	0.11	1.64	(1.23–2.19)	4.16 ± 0.36	4.15 ± 0.36	0.00	0.979	0.01	1.00	(0.76–1.32)
Push-up test (reps/min)	12.31 ± 11.23	10.32 ± 9.68	9.29	0.002	0.10	0.67	(0.52–0.87)	2.77 ± 5.12	2.50 ± 5.10	1.75	0.186	0.04	0.75	(0.48–1.15)
Horizontal jump (m)	1.61 ± 0.42	1.56 ± 0.35	3.45	0.063	0.06	1.39	(0.98–1.97)	1.33 ± 0.35	1.29 ± 0.31	1.61	0.204	0.04	1.36	(0.85–2.19)

BMI: Body mass index; ∑3 skinfolds: Sum of three skinfolds; PHV: Peak height velocity; CMJ: Counter movement jump.

**Table 5:** Odd ratio of suffering shoulder pain according to fitness and anthropometric variables in the general sample.

Variable	Group (*n* = 2015)		χ^2^	*p*	Cramer’s V	OR	95% CI
	Not Shoulder Pain (*n* = 1473)	Shoulder Pain (*n* = 542)					
	Mean ± SD	Mean ± SD					
Age (years)	14.35 ± 1.35	14.68 ± 1.45	1.20	0.273	0.02	0.90	(0.73–1.09
Physical activity level	2.72 ± 0.65	2.61 ± 0.65	3.76	0.052	0.04	1.05	(1.00–1.11)
BMI (kg/cm^2^)	21.18 ± 3.95	21.34 ± 3.52	4.56	0.033	0.03	1.06	(1.00–1.12)
Fat mass (% BIA)	23.85 ± 7.96	24.42 ± 7.21	0.85	0.356	0.02	0.87	(0.69–1.09)
Body mass (kg)	56.68 ± 13.06	57.25 ± 11.83	0.05	0.826	0.01	1.01	(0.95–1.06)
Height (cm)	163.24 ± 9.20	163.35 ± 8.81	1.87	0.172	0.03	0.96	(0.91–1.02)
Waist girth (cm)	69.46 ± 8.87	69.61 ± 8.34	1.42	0.233	0.03	1.03	(0.98–1.09)
Hips girth (cm)	90.35 ± 9.29	91.59 ± 8.16	13.63	0.000	0.08	1.45	(1.19–1.77)
∑3 skinfolds (mm)	50.82 ± 26.08	53.93 ± 24.22	9.24	0.002	0.07	1.36	(1.12–1.66)
Fat mass (% Slaughter)	22.25 ± 10.30	23.55 ± 9.70	6.37	0.012	0.05	1.07	(1.01–1.13)
Muscle mass (% Poortmans)	33.57 ± 5.61	32.16 ± 5.58	16.69	0.000	0.09	0.66	(0.54–0.80)
PHV (years)	0.36 ± 2.36	0.45 ± 2.65	5.05	0.025	0.05	1.27	(1.03–1.57)
20 m shuttle run test	4.13 ± 2.14	3.62 ± 1.96	23.29	0.000	0.11	0.60	(0.49–0.74)
VO2max (Legger’s formula)	38.48 ± 6.79	36.26 ± 6.64	27.75	0.000	0.12	0.58	(0.47–0.71)
CMJ (cm)	24.28 ± 6.69	24.01 ± 6.52	2.94	0.086	0.04	0.84	(0.69–1.03)
Sprint 20 m (s)	3.94 ± 0.42	3.97 ± 0.41	2.49	0.115	0.03	1.17	(0.96–1.43)
Push-up test (reps/min)	7.55 ± 9.72	5.83 ± 8.78	12.73	0.000	0.09	0.65	(0.51–0.82)
Horizontal jump (m)	1.44 ± 0.37	1.43 ± 0.40	2.91	0.088	0.04	1.29	(0.96–1.74)

BMI: Body mass index; ∑3 skinfolds: Sum of three skinfolds; PHV: Peak height velocity; CMJ: Counter movement jump.

**Table 6:** Odd ratio of suffering shoulder pain according to fitness and anthropometric variables in sample divided by sex.

Variable	Boys (*n* = 1006)							Girls (*n* = 1009)						
	Not Neck Pain (*n* = 798)	Neck Pain (*n* = 208)	χ^2^	*p*	Cramer’s V	OR	95% CI	Not Neck Pain (*n* = 681)	Neck Pain (*n* = 328)	χ^2^	*p*	Cramer’s V	OR	95% CI
	Mean ± SD	Mean ± SD						Mean ± SD	Mean ± SD					
Age (years)	14.32 ± 1.32	14.75 ± 1.44	0.73	0.394	0.03	0.88	(0.64–1.19)	14.39 ± 1.38	14.63 ± 1.46	0.97	0.325	0.03	0.88	(0.67–1.14)
Physical activity level	2.87 ± 0.63	2.78 ± 0.64	1.47	0.226	0.04	1.21	(0.89–1.65)	2.53 ± 0.62	2.51 ± 0.63	0.16	0.692	0.01	0.95	(0.72–1.25)
BMI (kg/cm^2^)	21.03 ± 3.97	21.87 ± 3.81	16.06	0.000	0.13	1.87	(1.37–2.54)	21.36 ± 3.92	21.00 ± 3.27	0.38	0.535	0.02	0.92	(0.70–1.20)
Body mass (kg)	58.50 ± 14.29	62.31 ± 12.98	8.44	0.004	0.09	1.58	(1.16–2.16)	54.54 ± 11.09	54.03 ± 9.77	0.96	0.328	0.03	0.87	(0.66–1.15)
Height (cm)	166.27 ± 9.95	168.07 ± 10.38	5.47	0.019	0.07	1.48	(1.06–2.07)	159.70 ± 6.67	160.32 ± 5.91	1.42	0.233	0.04	0.84	(0.63–1.12)
Waist girth (cm)	71.37 ± 9.13	73.53 ± 8.32	12.84	0.000	0.12	1.77	(1.29–2.42)	67.21 ± 8.00	67.09 ± 7.33	0.01	0.907	0.01	1.02	(0.76–1.35)
Hips girth (cm)	89.29 ± 9.69	91.82 ± 8.82	16.05	0.000	0.13	1.87	(1.37–2.54)	91.60 ± 8.63	91.44 ± 7.71	0.66	0.418	0.03	1.12	(0.86–1.45)
∑3 skinfolds (mm)	41.83 ± 24.76	44.34 ± 25.47	1.69	0.193	0.04	1.25	(0.89–1.75)	61.39 ± 23.51	60.10 ± 21.25	0.25	0.620	0.02	1.07	(0.82–1.40)
Fat mass (% Slaughter)	18.78 ± 10.24	20.16 ± 11.00	1.70	0.193	0.04	1.24	(0.90–1.72)	26.31 ± 8.78	25.73 ± 8.05	0.00	0.974	0.01	1.00	(0.76–1.30)
Muscle mass (% Poortmans)	37.33 ± 4.34	37.28 ± 4.55	0.13	0.716	0.01	1.08	(0.72–1.62)	29.15 ± 3.18	28.86 ± 3.19	0.11	0.745	0.01	1.08	(0.68–1.71)
PHV (years)	−0.19 ± 2.29	0.00 ± 2.53	3.29	0.070	0.06	1.33	(0.98–1.80)	0.99 ± 2.28	0.74 ± 2.69	2.25	0.134	0.05	0.78	(0.57–1.08)
20 m shuttle run test	5.08 ± 2.16	4.83 ± 2.06	3.39	0.066	0.06	0.75	(0.55–1.02)	2.96 ± 1.41	2.81 ± 1.38	3.10	0.078	0.05	0.74	(0.53–1.04)
VO2max (Legger´s formula)	41.21 ± 6.58	39.33 ± 6.95	3.64	0.056	0.06	0.73	(0.53–1.01)	35.25 ± 5.48	34.24 ± 5.59	6.31	0.012	0.06	0.67	(0.49–0.92)
CMJ (cm)	27.23 ± 6.76	27.93 ± 7.03	0.14	0.704	0.01	0.95	(0.72–1.24)	20.82 ± 4.65	21.44 ± 4.62	0.01	0.935	0.03	1.01	(0.74–1.38)
Sprint 20 m (s)	3.74 ± 0.36	3.73 ± 0.40	0.07	0.791	0.01	1.05	(0.74–1.49)	4.17 ± 0.37	4.12 ± 0.33	3.12	0.077	0.06	0.78	(0.59–1.03)
Push-up test (reps/min)	11.59 ± 10.72	11.54 ± 10.73	1.35	0.246	0.03	0.83	(0.61–1.14)	2.71 ± 5.23	2.42 ± 4.86	0.60	0.439	0.02	0.83	(0.51–1.33)
Horizontal jump (m)	1.57 ± 0.39	1.65 ± 0.39	4.18	0.061	0.07	1.51	(1.02–2.24)	1.31 ± 0.31	1.30 ± 0.35	1.95	0.163	0.04	1.39	(0.87–2.22)

BMI: Body mass index; ∑3 skinfolds: Sum of three skinfolds; PHV: Peak height velocity; CMJ: Counter movement jump.

## Data Availability

The data presented in this study are available on request from the corresponding author on reasonable request.
